# Decision-making during NHEJ: a network of interactions in human Polμ implicated in substrate recognition and end-bridging

**DOI:** 10.1093/nar/gku475

**Published:** 2014-05-31

**Authors:** Maria Jose Martin, Luis Blanco

**Affiliations:** Centro de Biologia Molecular Severo Ochoa (CSIC-UAM), 28049 Madrid, Spain

## Abstract

Human Polμ is a DNA polymerase belonging to the X family that has been implicated in the non-homologous end-joining (NHEJ) pathway during repair of double-strand breaks in DNA. Loop1 is a flexible piece of Polμ which has a critical role during terminal transferase and end-joining activities: it acts as a pseudo-template when the template strand is discontinuous or unavailable, whilst diffusing away if present to avoid steric clashes. Mutational analysis and inspection of the 3D structures available allowed us to identify a network of residues in charge of sensing the presence or absence of discontinuities in the template strand, which will in turn determine the final position adopted by Loop1. This network is formed by the previously uncharacterized thumb mini-loop (NSH motif) and the positively charged helix N, which contribute to the correct positioning of Loop1 and to juxtapose the discontinuous template strand during NHEJ of incompatible ends. Accordingly, single mutation of specific conserved residues in these motifs, whilst irrelevant in most of the cases for gap filling, largely affected terminal transferase and end-joining activities. Other point mutations in the ‘hinges’ of Loop1, such as residues Phe^385^ or Phe^389^, corroborated the flexibility requirements of this motif.

## INTRODUCTION

Template instruction is a general feature of most members of the human X family of polymerases, with the exception of Terminal Deoxynucleotidyl Transferase (TdT). TdT is the only known deoxyribonucleic acid (DNA) polymerase that is strictly DNA template independent, as it is able to add nucleotides to a DNA primer molecule only in the absence of a template chain. This feature is crucial for its function in V(D)J recombination, where TdT generates variability as it creates new information (1–3). Interestingly, Polμ shows hybrid biochemical properties: it has an intrinsic terminal transferase activity, but it is strongly activated by a template DNA chain to insert both deoxy- and ribonucleotides (4–7).

The structural and functional basis of the template independence of TdT was understood with the resolution of the crystal structure of the Polß-like core of TdT (8). A loop region between β-strands 3 and 4, referred to as Loop1, has a similar position in all three TdT structures and is located in a region of the DNA binding cleft that would normally be occupied by the template strand. Therefore, this loop could occlude binding of any DNA substrate possessing a template strand, explaining TdT inactivity on these substrates. On that basis, and by extrapolation of the structural model of TdT to Polμ, it was predicted that Loop1, specifically present in these two enzymes, could be directly responsible for their template-independent terminal transferase activity. In Polμ, however, Loop1 must be flexible enough to allow template-directed polymerization, being fully dispensable for gap filling (9). In agreement with this prediction, when the crystal structure of Polμ bound to a gaped DNA was solved (10), the DNA duplex was bound in the usual fashion within the DNA binding cleft, whereas Loop1 was disordered suggesting conformational flexibility. It was then clear that Polμ Loop1 cannot occupy the same position as that of TdT when a template strand is present. A comparison of the ends of the ß-strands flanking the loop shows that TdT's Loop1 extrudes towards the DNA binding cleft, whilst that of Polμ appears to turn away from the cleft. Although no crystal structure is available of Polμ with a single-stranded or 3′-protruding DNA substrate, it is likely that Loop1 would then be found in the same conformation as in TdT, i.e. interacting with the primer strand, somehow mimicking a ‘pseudo’ template strand. The structural evidence suggested that Loop1 in Polμ may adopt different conformations depending on the nature of the substrate. Studies including the Loop1 chimeras of Polμ (9) and TdT (11) confirmed this hypothesis: replacement of the TdT Loop1 with that of Polμ is sufficient to allow template-dependent additions, whilst the reciprocal chimera (Polμ with the TdT Loop1) is much less inclined to perform template-dependent additions. The importance of this structural feature is emphasized by its possible role as a regulatory element of the most mutagenic Polμ activities during the S phase of the cell cycle (12).

The equivalent regions in Polß and Polλ would be less likely to interfere with binding of the template strand because they have a much shorter Loop1: small enough in Polß to be described as a turn, and of intermediate length in Polλ. When Loop1 in Polμ is shortened to a length similar to that of Polλ, the altered polymerase has higher catalytic efficiency on template-containing substrates, but is incapable of template-independent synthesis (9,13). Consistent with all this, Polλ has strongly reduced ability to catalyze template-independent synthesis, but retains the ability to perform template-instructed additions. Polλ Loop1 may be involved in a function somehow related to that in Polμ: modulation of fidelity by controlling deoxynucleoside triphosphate (dNTP)-induced movements of the template strand and 3′-primer terminus in the transition from an inactive to an active conformation of the enzyme (14).

Both Polμ and Polλ have been involved in the non-homologous end-joining (NHEJ) pathway of DNA repair. Compelling evidence indicates that the NHEJ pathway minimizes loss of genetic material by using any template available (15–18). To achieve this, highly specialized polymerases perform ‘alignment-based gap fill-in’ by dealing with two DNA ends: one providing the primer (a protruding 3′-OH) and a second DNA molecule (with a recessive 5′-P and a 3′-protruding template strand) providing a template *in trans* (the present study does not focus on blunt or 5′-protruding ends since these do not require specialized polymerases to be repaired). Our previous studies indicate that one Polμ monomer is able to bind both sides of the break at once (19). But the opportunity to act during NHEJ is dictated by the enzyme's template preference, a property that follows a gradient ranging from Polß, which only polymerizes on substrates with a continuous template strand; to Polλ, which is active in NHEJ only when the template strand is stabilized by complementarity with the primer strand; to Polμ, which can direct template-instructed primer extension even when there is no base pairing between the two ends; to TdT, which also acts on unpaired primer termini but does not allow the use of a template strand [reviewed in (20)]. It has been suggested that this variable degree of template dependence relies on structural differences amongst the four polymerases. In this work, we have deciphered some of these structural determinants, in particular a network of interactions for substrate recognition and end-bridging, conferring Polμ the unique handiness of *trans*-polymerizing without the help of a single base pair connection amongst the two DNA ends. This network is formed by the previously uncharacterized thumb mini-loop (NSH motif) and the positively charged helix N. The role of Loop1 during these nucleotide additions has been treated in depth here, including a highly detailed study of how Polμ fixes and/or orients this mobile part of the protein in accordance with the substrate on which it is polymerizing.

## MATERIALS AND METHODS

### DNA and proteins

Synthetic DNA oligonucleotides were obtained from Isogen (Ijsselstein, Holland). Polyacrylamide gel electrophoresis (PAGE)-purified oligonucleotides were labelled at their 5′-ends with [γ-^32^P]ATP. The oligonucleotides used to generate the DNA substrates were the following: for 1-nt gaped substrates, Sp1C (5′GATCACAGTGAGTAC), T13C (5′AGAAGTGTATCTCGTACTCACTGTGATC) and DG1P (5′AGATACACTTCT). For NHEJ assays, three sets of oligonucleotides were used. A first set of primers sharing the same common part (5′CCCTCCCTCCC…) and bearing different 3′ protrusions (1AC […CA3′], 1TG […GT3′], 1A […A3′], 1C […C3′], 1G […G3′], 1T […T3′]) was hybridized to 1D-NHEJ (5′GGGAGGGAGGG). A second set of primers sharing the common part (5′GCACTCACGTCCC…) and bearing different 3′-overhangs (2AC […CA3′], 2TG […GT3′], 2A […A3′], 2C […C3′], 2G […G3′], 2T […T3′]) was hybridized to oligonucleotide 2D-NHEJ (5′GGGACGTGAGTGC). Oligonucleotides DG1P and 2D-NHEJ contain a 5′P group. Ultrapure dNTPs, ddNTPs, [α-^32^P] dNTPs (3000 Ci/mmol) and [γ-^32^P]ATP (3000 Ci/mmol) were purchased from GE Healthcare (UK). T4 polynucleotide kinase was obtained from New England Biolabs (Beverly, MA, USA). Pfu DNA polymerase was purchased from Promega Corporation (Madison, WI, USA).

### Construction and purification of human Polμ mutant proteins

Site-directed mutagenesis by single polymerase chain reaction with oligonucleotides containing the desired mutation was performed on the Polμ-over-expressing plasmid pRSETA-hPolμ (21). The oligonucleotides used were the following: F385G (5′CACATGGACGCTGGTGAGAGAAGTTTC), F389G (5′TTTGAGAGAAGTGGCTGCATTTTCCGC), F389L (5′TTTGAGAGAAGTTTATGCATTTTCCGC), R442A (5′AAGCTTTTCCAGGCGGAGCTGCGCCGC), R442K (5′AAGCTTTTCCAGAAGGAGCTGCGCCGC), R445A (5′CAGCGGGAGCTGGCGCGCTTCAGCCGG), R449G (5′CGCCGCTTCAGCGGGAAGGAGAAGGGC), N457D (5′GGCCTGTGGCTGGACAGCCATGGGCTG), S458N (5′CTGTGGCTGAACAACCATGGGCTGTTT) and H459G (5′TGGCTGAACAGCGGTGGGCTGTTTGAC). DNA constructs were sequenced and transformed in *Escherichia coli* BL21(DE3)pLysS. Wild-type and mutant Polμ variants were over-expressed and purified in an Äkta Purifier FPLC system (GE Healthcare) with the following protocol: the cleared bacterial lysate was loaded on a heparin column followed by an S sepharose column. The selected fractions were then loaded on a HiPrep Sephacryl 26/60 to eliminate contaminant nucleases. The eluted fractions containing highly purified protein were concentrated and stored at −80°C.

### DNA polymerization and NHEJ assays

DNA substrates, containing 5′P-labelled primers (1 nM), were incubated for 30 min at 30°C with the indicated amounts of enzyme. When stated, an excess of cold substrates was added to the reactions (6 nM). The reaction mixture, in 20 μl, contained 50-mM Tris-HCl (pH 7.5), 1-mM DTT (dithiothreitol), 4% glycerol and 0.1-mg/ml bovine serum albumin, in the presence of the indicated amounts of the DNA polymerization substrates, and the indicated concentrations of dNTPs and activating metal ions. After incubation, reactions were stopped by adding gel loading buffer [95% (v/v) formamide, 10-mM EDTA (ethylene diamine tetraacetic acid), 0,1% (w/v) xylene cyanol and 0.1% (w/v) bromophenol blue] and analysed by 8-M urea/20% PAGE and autoradiography. When indicated, we used ddNTPs instead of dNTPs to limit incorporation to a single nucleotide on the 3′-end of the labelled oligonucleotide.

### Amino acid sequence comparisons and 3D modelling

Multiple alignment of different DNA polymerases was done using the program MULTALIN (http://prodes.toulouse.inra.fr/multalin/). The different conformations of the studied residues, motifs and domains in the X family polymerases were analysed with the software MacPymol (http://delsci.com/macpymol/).

## RESULTS

### Loop1 confers Polμ its NHEJ efficiency on short 3′-overhangs

**Figure 1.**
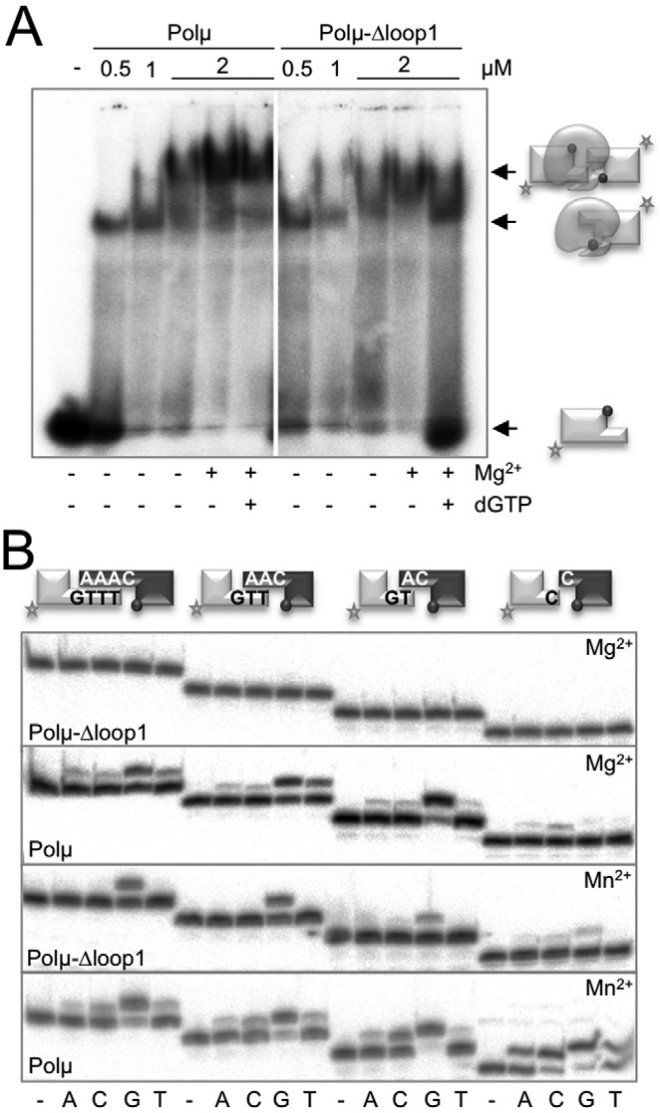
Loop1 confers Polμ its NHEJ efficiency on short 3′-overhangs. (**A**) EMSA was performed for the wild-type and mutant Polμ at the indicated amounts (0.5, 1, 2 μM) using a 3′-protruding substrate (1 nM) containing oligonucleotides 1TG and 1D-NHEJ. When indicated, 2.5-mM MgCl_2_ and/or 10-μM dGTP were added. After electrophoresis, gel was dried and the labelled fragments were detected by autoradiography. (**B**) NHEJ reactions were performed with 200 nM of the indicated proteins using four sets of substrates: the labelled substrates were formed by hybridization of 1TTTG, 1TTG, 1TG or 1C with 1D-NHEJ, and the cold substrates by hybridization of either 1AAAC, 1AAC, 1AC or 1C with 1D-NHEJ. The grey spheres indicate the presence of a 5′-P group in the downstream strand of the substrate. When indicated, each of the four ddNTPs (10 μM) was added in the presence of 2.5-mM MgCl_2_ or 1-mM MnCl_2_.

Loop1 is a specific subdomain in Polμ, shared with TdT, which is flexible and thus can adopt multiple conformations, and probably acts as a pseudo-template when a proper template DNA strand is not available for instructing polymerization. This subdomain could be resolved in the crystal structure of TdT, but not in that of Polμ. Supplementary Figure S1A shows a superimposition of the conformation adopted by Loop1 of TdT in the murine apoenzyme (PDB ID: 1JMS), modelled on the ternary structure of murine Polμ with gaped DNA and incoming nucleotide (PDB ID: 2IHM, wheat). In agreement with its location in the core structure, Loop1 has been implicated in the terminal transferase activity of human Polμ (9) and in NHEJ of non-complementary ends assisted by accessory factors (13). To corroborate the importance of Loop1 for the bridging activity inherent to human Polμ we tested a Loop1-deletion mutant [Polμ-Δloop1; (9)] that lacks amino acids 369 to 385. It has been previously shown that binding of 5′P-containing gaped substrates by Polμ-Δloop1 is even higher than that displayed by the wild-type enzyme, suggesting that Loop1 is dispensable and even detrimental for binding to template-containing substrates perhaps via steric hindrance (9). Wild-type Polμ can form two different complexes when binding a 5′P-containing 3′-protruding substrate (Figure 1A). The first shifted band corresponds to the binding to one DNA molecule (with the protrusion oriented as template), whilst the second band likely represents the synapsis of two DNA molecules (Figure 1A). Mutant Polμ-Δloop1 was able to bind the downstream side of the break (Figure 1A); however, the synapsis could not be stably formed in the absence of Loop1, not even in the presence of metal and incoming nucleotide (Figure 1A). Moreover, the activity of this mutant was undetectable in NHEJ assays, both with complementary and non-complementary ends, even using substrates that can form two and three base pairs of complementarity (Figure 1B, top panel). Having shown that Loop1 is dispensable for binding the 5′P-containing DNA as a downstream end, the defective NHEJ reaction observed can only be explained if Loop1 plays a role in the correct juxtaposition of the incoming end acting as a primer, in line with the EMSA (Electrophoretic Mobility Shift Assay) results. NHEJ by this mutant was partially recovered when using manganese as activating metal ion. However, we realized that the substrate preference for the mutant lacking Loop1 changes with respect to the wild-type enzyme: the mutant version achieves better NHEJ mediated by longer connections (Figure 1B, lower panel; as would be expected due to the help of complementary base pairing), whilst wild-type Polμ strikingly prefers the shortest 3′-overhangs over longer ones, even in the presence of manganese (3 and 4 nts; Figure 1B, middle and lower panels). Therefore, Loop1 appears to be conferring the unique Polμ property of bridging ends with very short overhangs. This observation also implies that a minimal distance between the two gaps formed during end joining allows the optimal location, and thus performance, of Loop1. This proposal is supported by the available crystal structures of TdT and Polμ. Supplementary Figure S1B and C show two different versions of the superimposition described above: in (B), the DNA substrate has been modified to show a 2-nt 3′-protruding substrate bound as a template/primer, a scenario in which Loop1 would help to maintain the orientation of the primer strand to achieve the untemplated addition of an incoming nucleotide, shown in blue; in (C), the original DNA substrate is lacking template bases A6 and T7 in order to mimic two non-complementary NHEJ substrates: the ‘template end’ having a 3′ overhang of 1 nucleotide (purple) and the ‘primer end’ with a 2-nt 3′-protrusion (green). As we show here, Loop1 would fit in the space corresponding to -1 and -2 positions of a continuous template strand, and, therefore, it could be conveniently positioned to help preferential bridging of NHEJ substrates with very short overhangs.

### Relationship between TdT activity and NHEJ efficiency: single mutations in Loop1 affecting its structure/function

**Figure 2.**
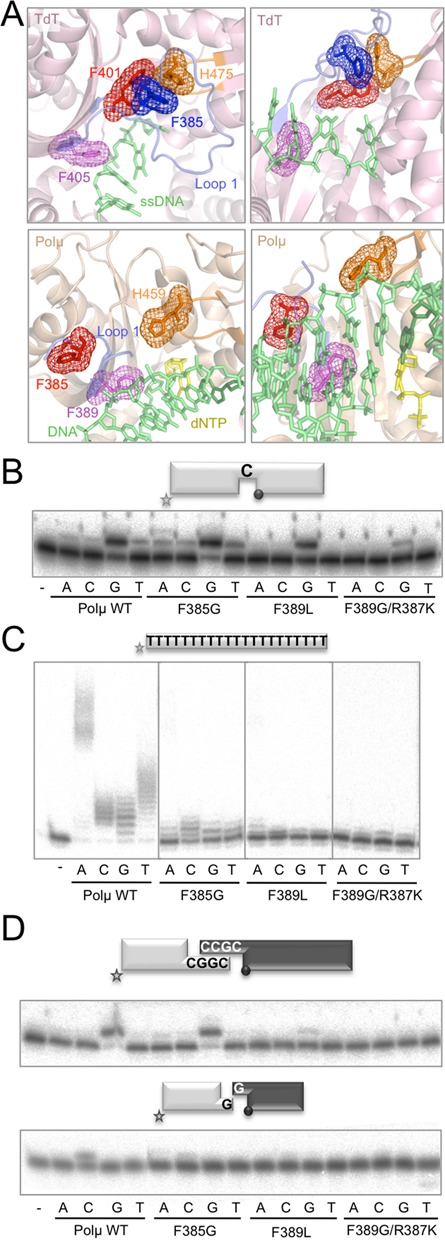
Relationship between TdT activity and NHEJ efficiency: single mutations in Loop1 affecting its structure/function. (**A**) Cartoon representations of the structures of murine TdT bound to ssDNA (1KDH, light pink) and the murine Polμ ternary complex (2IHM, wheat), showing the Loop1 in a blue cartoon and selected residues in sticks and mesh. Numbering of Polμ residues corresponds to the human enzyme, for congruence with the numbering used throughout the text. DNA substrate is shown in green and incoming nucleotide in yellow. (**B**) Gap-filling reactions were performed as described in the Materials and Methods section with the indicated proteins (25 nM) using a gaped substrate containing the oligonucleotides SP1C, T13C and DG1-P. When indicated, dNTPs were added separately at 10 nM in the presence of 2.5-mM MgCl_2_. (**C**) Terminal transferase activity assay with the indicated proteins (600 nM) using a homopolymeric substrate (polydT) and each of the four dNTPs (100 μM). Reactions were incubated for 30 min at 37ºC. (**D**) NHEJ reactions were performed with 200 nM of the indicated proteins and using four sets of substrates: the labelled substrates were formed by hybridization of 1G with 1D-NHEJ or D3-C with D1, and the cold substrates by hybridization of either 2G with 2D-NHEJ or D4-C with D2. The gray spheres indicate the presence of a 5′-P group in the downstream strand of the substrate. When indicated, each of the four ddNTPs (10 μM) was added in the presence of 2.5-mM MgCl_2_.

Once we have shown the importance of Loop1 in NHEJ reactions performed by Polμ in the absence of accessory factors, we studied the mechanism of action of this motif by mutational analysis of candidate residues to be involved in specific interactions with the DNA substrates. Guided by protein multi-alignments of the four family X members (Supplementary Figure S2A) and by comparison of the available crystal structures (Figure 2A) of the murine TdT (PDB IDS: 1KDH, 1JMS) and Polμ (PDB ID: 2IHM, monomers A and B), we decided to mutate three human Polμ residues included in or near Loop1: Phe^385^, Arg^387^ and Phe^389^.

In TdT (Figure 2A, top panels: left panel for top view, right panel for frontal view) Phe^401^ (corresponding to Phe^385^ in Polμ, red), located in the border region of Loop1, is interacting with another highly conserved phenylalanine in the middle of this motif in TdT (Phe^385^, blue), which seems to be involved in maintaining the fixed position of Loop1 via a strong stacking interaction between its aromatic ring and His^475^ (His^459^ in Polμ, orange) at the thumb subdomain. Recent data have shown that mutation of Phe^401^ to alanine in TdT strongly reduced the terminal transferase activity of the polymerase and, strikingly, allowed templated addition of nucleotides, turning a completely template-independent enzyme into a DNA-instructed DNA polymerase (11). Our explanation of these results is that by mutating this residue the network of interactions needed to maintain a fixed orientation of TdT Loop1 is abolished, and Loop1 is now endowed with a greater degree of flexibility, as in Polμ, thus allowing TdT to accept a template strand. Despite the sequence conservation of this phenylalanine between TdT (Phe^401^) and Polμ (Phe^385^), there might not be a functional conservation, since the second phenylalanine involved in TdT (Phe^385^) is not present in Polμ (Figure 2A, bottom panels: left for top view, right for frontal view). To investigate this difference, we decided to mutate Phe^385^ in Polμ to glycine in order to establish its putative role in the terminal transferase and NHEJ activities of Polμ. As a control, we firstly confirmed that elimination of the aromatic ring (by substitution of Phe^385^ into a glycine) does not affect Polμ's gap-filling activity (Figure 2B). Terminal transferase activity of mutant F385G, on the other hand, was largely abolished when compared to wild-type Polμ (Figure 2C), thus confirming that this residue has a specific role in the catalytic cycle only when a template strand is not available. Next, we tested this mutation on NHEJ assays, in which the DNA substrates contain discontinuities not only in the primer strand but also in the template strand in a position that Loop1 might help to coordinate. Interestingly, the activity of the enzyme was lower than that of the wild-type Polμ, but only on non-complementary ends (Figure 2D, bottom panel), whose end-joining is a hallmark of Polμ.

Phe^389^ is specifically conserved amongst Polμs and TdTs (Phe^405^ in murineTdT) of different species (Supplementary Figure S2A), and in both cases it could be involved in maintaining the structure of the bordering region of Loop1, thus probably affecting the shape and orientation of this motif (Figure 2A, purple). Mutation of this residue (Phe^405^) to alanine in murine TdT abolishes terminal transferase activity and allows templated insertion of only one nucleotide on a template/primer substrate (11). We decided to mutate the equivalent human Polμ residue (Phe^389^) to leucine (the amino acid present in human Polλ and in some Polμs from other species) to demonstrate its importance during the catalytic cycle of human Polμ. We also made the double mutation F389G/R387K, which could be expected to have a boosted terminal transferase activity [single mutant R387K increases this activity by up to 100-fold (3)]. Both mutants were tested for gap-filling activity and, as showed in Figure 2B, the double mutant was affected when compared to the wild-type Polμ. The expected implication of Phe^389^ in the ability of Polμ to catalyze untemplated nucleotide additions was confirmed by testing the terminal transferase activity of these mutants: it was drastically abolished (Figure 2C), also in the case of the double mutant, which is specially noticeable since this enzyme also contains the R387K mutation (3). When NHEJ activity was assayed (Figure 2D), the two mutants were completely negative, as expected from an affected orientation of Loop1 and the consequent lack of coordination of the DNA substrates during this kind of reactions.

### The thumb mini-loop: flexibility of Loop1

By analysing the available structures of TdT (PDB IDs: 1JMS, 1KEJ and 1KDH) we noticed that a second loop, located in the thumb subdomain, is establishing interactions with Loop1. This thumb ‘mini-loop’ contains several conserved residues responsible for these interactions, amongst them the invariant Asp^473^, Asn^474^ and His^475^ in murine TdT (Supplementary Figure S2B, DNH motif). This thumb mini-loop is also present in Polμ, but the sequence is not strictly conserved (Supplementary Figure S2B, NSH motif). The only invariant residue is the histidine, His^459^ in human Polμ. Whereas the other two residues present in the murine TdT sequence, Asp^473^ and Asn^474^, are an asparagine (Asn^457^) and a serine (Ser^458^), respectively, in human Polμ (Supplementary Figure S2B).

**Figure 3.**
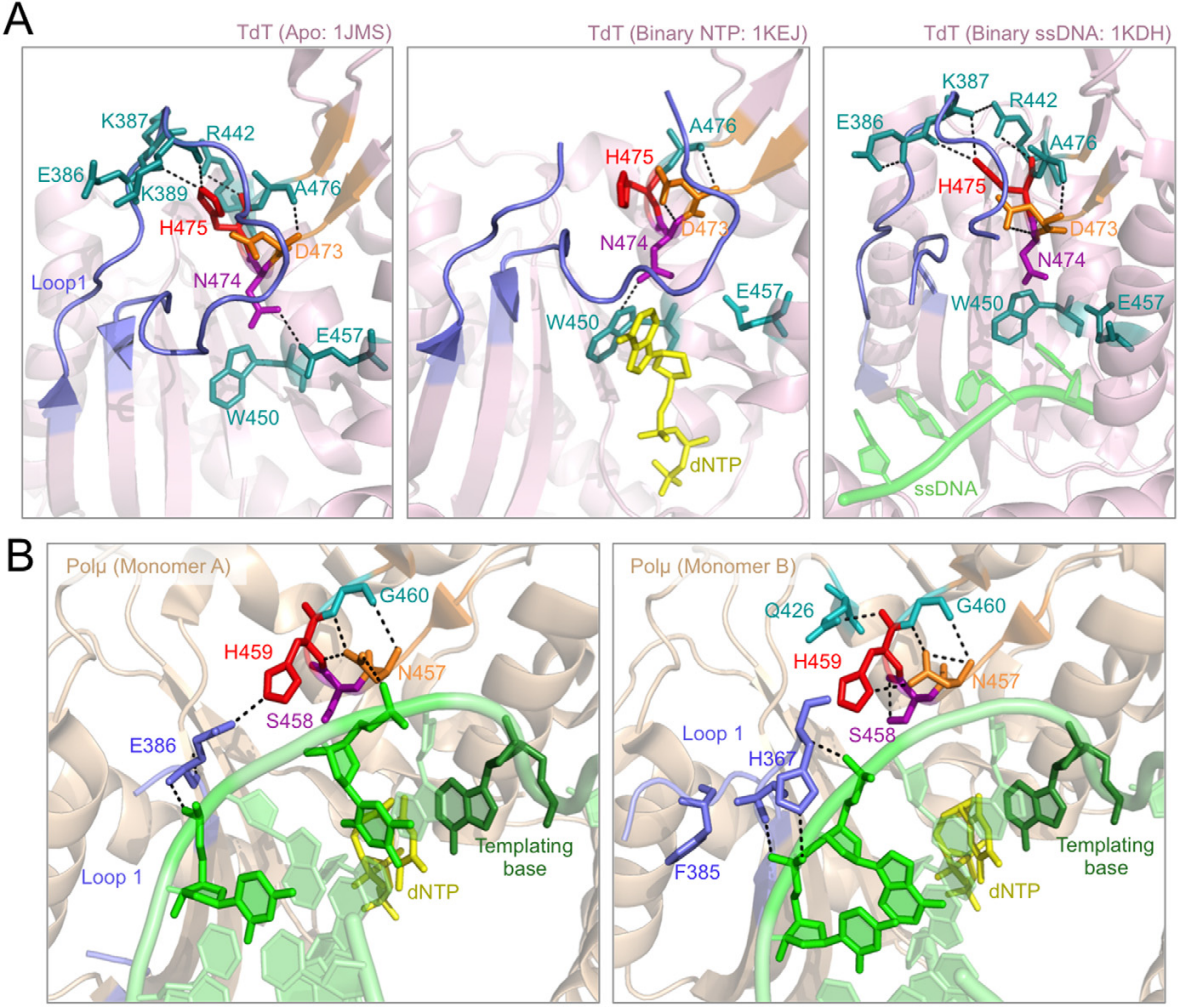
The thumb ‘mini-loop’ (NSH motif). (**A**) Cartoon representation of the three available TdT structures showing Loop1 in a blue cartoon and the thumb ‘mini-loop’ in an orange cartoon. Selected residues are shown in orange, red and purple sticks, whilst the residues included in their network of contacts are shown in teal-coloured sticks. Incoming dNTP is shown in yellow sticks, DNA substrate is shown in green sticks. (**B**) Cartoon representation of the two monomers included in the Polμ crystal structure (2IHM) showing Loop1 in a blue cartoon and the thumb ‘mini-loop’ in an orange cartoon. The mutated residues are shown in orange, red and purple sticks, whilst the residues included in their network of contacts are shown in teal-coloured sticks. Numbering of Polμ residues corresponds to the human enzyme. Incoming dNTP is shown in yellow sticks, DNA substrate is shown in green sticks.

In TdT, Asp^473^ seems to be involved in maintaining the general conformation of this mini-loop through interactions with other residues in the motif (Asn^474^, Ala^476^; Figure 3A). Asn^474^ changes very slightly its orientation in the three TdT crystals available, but this minor movement is enough to allow different interactions in each case: in the Apo structure this residue is interacting with Glu^457^, a residue that is only at contact distance in the apoenzyme (Figure 3A, left panel); in the NTP-bound structure, Asn^474^ is contacting Trp^450^ probably due to its stacking against the incoming nucleotide that slightly affects its position (Figure 3A, central panel); finally, in the TdT-ssDNA co-crystal, this asparagine is not making any contacts, since none of its partners is available for interaction (Figure 3A, right panel). In TdT, His^475^ is establishing a strong network of direct interactions with Loop1, through residues Glu^386^ and Lys^387^ or Lys^389^, depending on the crystal structure studied. His^475^ also makes direct interactions with residue Arg^442^. There is one TdT crystal in which part of Loop1 (corresponding to the residues involved in these interactions) is not observed in the electron density: the binary complex with incoming nucleotide (Figure 3A, central panel), in which His^475^ has rotated and now this stabilizing network is disrupted.

All these observations led us to study the possible role of this thumb mini-loop in Polμ, which could also have a Loop1-stabilizing function, not as constitutively as in TdT, but perhaps specifically during NHEJ. The available Polμ structure in complex with a gaped substrate and incoming nucleotide (Figure 3B) includes two monomers in the unit cell, and the two of them show different states and interactions of residue His^459^ (the counterpart of His^475^ in TdT): in one of the monomers it is interacting with Glu^386^ from Loop1, perhaps mimicking the function of the His^475^/Phe^385^ pair in TdT, but in the other it is contacting Asn^457^ (the counterpart of Asp^473^ in TdT). This interaction between His^459^ and Asn^457^ seems to be capturing the histidine and preventing its interaction with Glu^386^ in Loop1, probably allowing it to be repositioned in a different conformation to allow binding of a template strand. When the template strand is not present (terminal transferase) or is discontinuous (NHEJ), His^459^ might help in stabilizing Loop1 through alternative interactions, in a position more similar to that observed in the TdT crystals. Interestingly, when His^459^ is interacting with Glu^386^, Asn^457^ directly contacts the template strand (Figure 3B, monomer A). Given the predicted importance of the NSH motif we obtained mutants H459G (to abolish the function), N457D, S458N, and the double mutation N457D/S458N in order to mimic the residues present in TdT in this area.

**Figure 4.**
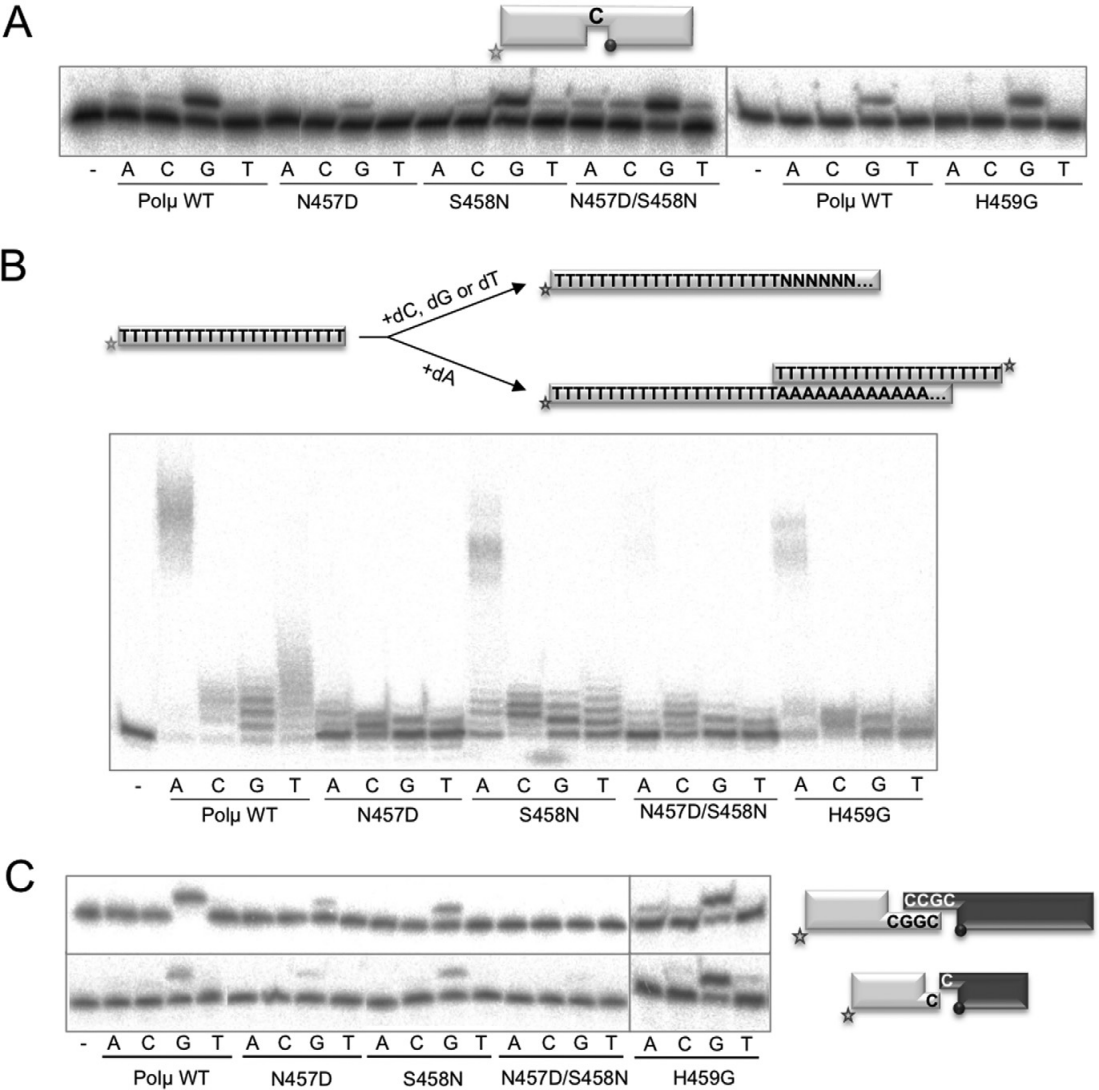
Mutations in the thumb ‘mini-loop’ (NSH motif) of human Polμ specifically affect terminal transferase and NHEJ. (**A**) Gap-filling reactions were performed as described in the Materials and Methods section with the indicated proteins (25 nM) using a gaped substrate containing the oligonucleotides SP1C, T13C and DG1-P. When indicated, dNTPs were added separately at 10 nM in the presence of 2.5-mM MgCl_2_. (**B**) Terminal transferase activity assay with the indicated proteins (600 nM) using a homopolymeric substrate (polydT) and each of the four dNTPs (100 μM). Reactions were incubated for 30 min at 37ºC. (**C**) NHEJ reactions were performed with 200 nM of the indicated proteins and using four sets of substrates: the labelled substrates were formed by hybridization of 1G with 1D-NHEJ or D3-C with D1, and the cold substrates by hybridization of either 2G with 2D-NHEJ or D4-C with D2. The black spheres indicate the presence of a 5′-P group in the downstream strand of the substrate. When indicated, each of the four ddNTPs (10 μM) was added in the presence of 2.5-mM MgCl_2_.

The behaviour of these mutants in gap-filling activity (i.e. in the presence of a continuous template strand) was different in each case: mutant N457D had lower activity than the wild type on this substrate, in agreement with its observed interaction with the template strand; S458N, double mutants N457D/S458N and H459G were able to perform gap filling as the wild-type Polμ (Figure 4A). When testing terminal transferase activity, mutant S458N was the only one that displayed a similar level of activity to that of the wild-type enzyme with any of the four dNTPs. Strikingly, both the single and double mutants having the change N457D were exclusively affected in the addition of dA nucleotide units, having wild-type levels of terminal transferase activity with dC, dG and dT (Figure 4B). The long (dA)n products produced by the wild-type Polμ have been interpreted mainly as the result of DNA-templated incorporation, allowed by the connection of a dA-extended PolydT (via terminal transferase) with another PolydT molecule, now acting as template [(9); see scheme in Figure 4B]. Therefore, it is very likely that mutation N457D is not affecting terminal transferase, but precludes the connection/synapsis step that is allowing template-directed incorporation of deoxyadenosine triphosphate (dATP). This implies that the DNA ligand function of Asn^457^ observed in a DNA gap is crucial for synapsis of complementary ends during NHEJ. Conversely, mutant H459G maintained normal levels of dA incorporation on PolydT, whilst addition of the other three dNTPs (via terminal transferase) was significantly inhibited. That would be in agreement with a role of His^459^ in maintaining the appropriate orientation of Loop1 for terminal transferase, but it appears not to be critical for the function of Loop1 during synapsis of two complementary ends.

To evaluate this hypothesis, we assayed these mutants on NHEJ reactions involving either complementary or non-complementary ends. Mutants N457D and S458N were significantly affected in NHEJ reactions involving complementary ends (N457D was also affected when non-complementary ends were tested), although maintained an error-free outcome (dG being preferentially inserted; Figure 4C). Interestingly, double mutant N457D/S458N, which had a wild-type behaviour on gap-filling reactions, was completely unable to perform NHEJ of both complementary and non-complementary ends. Residue His^459^ seems to be unnecessary since mutant H459G reached the same activity levels as the wild-type Polμ on both substrates tested (Figure 4C).

All these observations lead to the conclusion that this NSH motif (thumb mini-loop) in Polμ is playing a role during terminal transferase additions (mediated by His^459^), probably via stabilization of Loop1 in the absence of a template strand (thus mimicking the function of the DNH motif in TdT), and most specially during NHEJ reactions, probably by establishing interactions both with Loop1 and with the template strand that improve the connection of the two ends.

### The arginine helix facilitates template-dependent NHEJ

**Figure 5.**
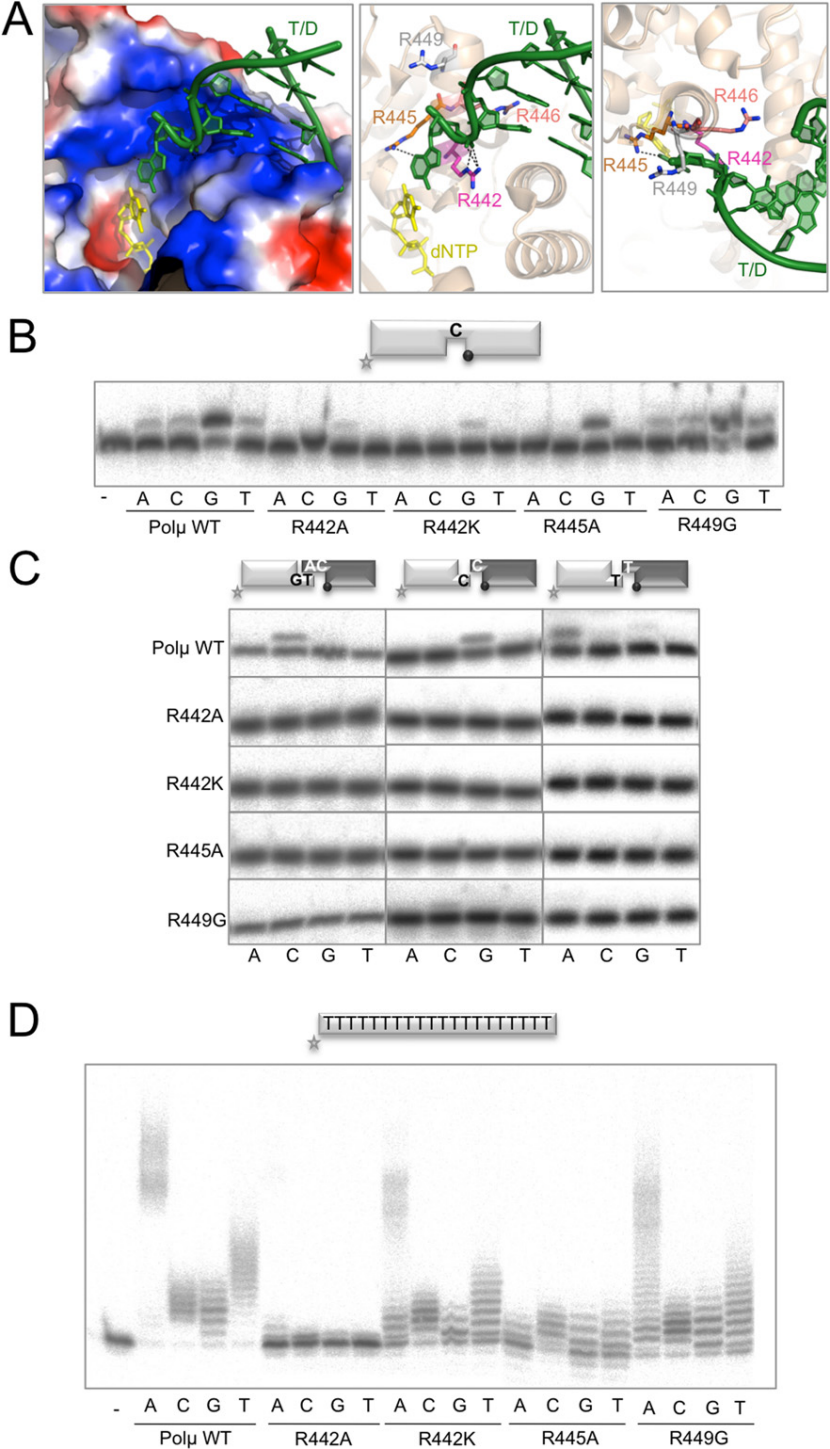
Residues implicated in binding the template strand. (**A**) Different representations of the Polμ ternary complex structure (2IHM) showing the region of helix N: electrostatic surface (left panel) showing the high amount of positive charge, and cartoon representations with the four arginines shown in sticks in side and top views (middle and left panels, respectively). Numbering of Polμ residues corresponds to the human enzyme. Incoming dNTP is shown in yellow and DNA substrate is shown in green. (**B**) Gap-filling reactions were performed as described in the Materials and Methods section with the indicated proteins (25 nM) using a gaped substrate containing the oligonucleotides SP1C, T13C and DG1-P. When indicated, dNTPs were added separately at 10 nM in the presence of 2.5-mM MgCl_2_. After electrophoresis, labelled fragments were detected by autoradiography. (**C**) NHEJ reactions were performed with 200 nM of the indicated proteins and using four sets of substrates: the labelled substrates were formed by hybridization of 1G with 1D-NHEJ or D3-C with D1, and the cold substrates by hybridization of either 2G with 2D-NHEJ or D4-C with D2. The black spheres indicate the presence of a 5′-P group in the downstream strand of the substrate. When indicated, each of the four ddNTPs (10 μM) was added in the presence of 2.5-mM MgCl_2_. (**D**) Terminal transferase activity assay with the indicated proteins (600 nM) using a homopolymeric substrate (polydA) and each of the four dNTPs (100 μM). Reactions were incubated for 30 min at 37ºC. After electrophoresis, labelled fragments were detected by autoradiography.

We next focused on a positively charged α-helix in human Polμ, which contains four arginines (Arg^442^, Arg^445^, Arg^446^ and Arg^449^) oriented towards the negatively charged phosphate backbone of the template strand (Figure 5A). Arg^442^ and Arg^445^ (present in Polμ, TdT, Polλ and Polß enzymes from different species; Supplementary Figure S2C) are analogous to Arg^514^ and Arg^517^ in human Polλ, which trigger both the DNA motion and the thumb loop motion (22), Arg^517^ also controlling fidelity at least in *in silico* simulations (23). In human Polß, Arg^283^ (analogous to Polλ Arg^517^ and Polμ Arg^445^) is also important for fidelity (24–26). Arg^442^ and Arg^445^ in Polμ interact with the DNA template through a series of hydrogen bonds and stacking interactions (Figure 5A), in a similar manner to the interactions established in Polλ. This may suggest the importance of Arg^442^ and Arg^445^ in maintaining the active (ternary) form of Polμ, via stabilization of the DNA template, especially in those situations in which the template strand is discontinuous. Arg^445^ may participate in the active-site assembly when an incorrect nucleotide exists at the active site, as suggested by the similar fidelity checking function of Arg^517^ in Polλ or Arg^283^ in Polß. In a recent publication (27), molecular dynamics simulations of Polμ showed that Arg^445^ affects the conformation of Loop1, and thus may be important for maintaining the Loop1–DNA interactions that are crucial for template-independent synthesis (9). Polμ Arg^446^ is not conserved in Polß or Polλ, where an alanine occupies the corresponding location, but it is conserved in TdT. This residue is implicated in maintaining a closed conformation of the polymerase core via interaction with the ‘brooch’, a 5-residue structure N-terminal to the 8-KDa domain (28). Arg^449^ is interacting with the template strand and is strictly conserved only amongst Polμs of different species, and not in the other three members of the family, and thus might have a special role in Polμ-specific functions. Taking all these observations into account, we decided to prepare the mutants R442A, R442K, R445A and R449G in order to determine how these residues contribute to the function of Polμ.

Figure 5B shows the activity of either wild-type or mutant Polμ during polymerization on a 1-nt gap. As expected, mutants in Arg^442^ were affected in their activity levels, whilst mutants in Arg^445^ and Arg^449^ displayed wild-type activity on this substrate. We then tested our hypothesis that the template-stabilizing function of these residues could be decisive when dealing with NHEJ substrates. As shown in Figure 5C, all the mutants were completely negative for end-joining reactions, either with complementary ends or with two different non-complementary ends, providing either a strong (C) or a weak (T) templating base (5), a situation in which the terminal transferase activity of Polμ can contribute to create connectivity between the two ends. These results clearly emphasize the need for a perfectly orchestrated synapsis, in which all the elements (two DNA ends and incoming nucleotide) must be in proper register for catalysis.

In order to analyse if these residues are selectively implicated in orientation of the template strand or whether they could also be implicated in interactions with other DNA substrates or amino acid motifs in the polymerase, the mutants were tested for terminal transferase addition of nucleotides on a homopolymeric single-stranded DNA (ssDNA) substrate (poly-dA). Strikingly, mutants R442A and R445A showed very low or undetectable levels of terminal transferase activity, in the presence of any of the four dNTPs, in comparison to the wild-type Polμ (Figure 5D). On the other hand, mutant R442K, in which the charge of the residue is conserved but not its shape or length, and R449, which is the residue located further away from the substrate, still displayed some terminal transferase activity.

A plausible explanation for the strong phenotype of mutants R442A and R445A in a reaction not involving a template strand could be their implication in stabilizing Loop1 as a template-mimicking structure, as already predicted in the molecular dynamics studies for Arg^445^ (27). In agreement with this hypothesis is the observation that Arg^442^ and Arg^445^ are conserved in TdT (Arg^458^ and Arg^461^), and even though in the crystal structures available there is no direct interaction between any of these arginines and Loop1, they are forming part of a network of interactions probably affecting the final position and orientation of Loop1. As described above, there is a mini-loop located in the thumb subdomain of TdT and Polμ, which appears to be implicated in stabilizing Loop1 in the position of the missing template strand. Interestingly, Polμ residues Arg^446^ and Arg^449^ interact through water molecules with residue Asn^457^ from the thumb mini-loop, which in turn interacts with His^459^ that is contacting Phe^385^ and/or Glu^386^, at the base of Loop1 in Polμ (Supplementary Figure S3, left panel). It is worth noting that in TdT a similar network is also found, as shown in Supplementary Figure S3, right panel.

## DISCUSSION

One of the structural features that is crucial for Polμ function in NHEJ is a mobile domain, named Loop1, whose 3D analysis is still insufficient due to the lack of a crystal structure corresponding to an NHEJ intermediate. Conversely, several crystal structures of TdT (the closest Polμ homologue) either as apoenzyme or bound to ssDNA and nucleotide substrates allowed visualization of Loop1 in a stabilized position that would interfere with the binding of a template DNA strand, but compatible with its importance for terminal transferase activity (8). In this study, we have made point mutations in human Polμ residues Phe^385^ and Phe^389^, located at the bordering regions of Loop1, guided by their conservation at the primary sequence level and by comparison of the available crystal structures of TdT and Polμ. Phe^401^ of TdT (corresponding to Phe^385^ in Polμ) is involved in maintaining the fixed position of Loop1 via a strong stacking interaction between its aromatic ring and His^475^ (His^459^ in Polμ), located in a mini-loop at the thumb subdomain. Mutant F401A in TdT had a striking phenotype, turning a completely template-independent enzyme into a DNA-instructed DNA polymerase (11). This mutation clearly disrupted the network of interactions needed to maintain a fixed orientation of TdT Loop1, now endowed with a greater degree of flexibility as it is in Polμ, thus allowing TdT to accept a template strand. Mutation F385G in Polμ, whilst not affecting templated additions, largely abolishes the terminal transferase activity of Polμ, thus confirming that Phe^385^ has a specific role in the catalytic cycle only when a template strand is not available. Similar results were reached by Moon *et al.* by mutating Phe^385^ to alanine in a recent study (29). Phe^389^ is again conserved amongst Polμs and TdTs (Phe^405^) of different species, and in both cases it seems to be involved in maintaining the shape and orientation of Loop1. Mutation of this residue to alanine in TdT abolished terminal transferase activity and allowed templated insertion of only one nucleotide on a template/primer substrate (11). The expected implication of Phe^389^ (the equivalent Polμ residue) in the ability of Polμ to catalyze untemplated nucleotide additions has been confirmed here by testing the terminal transferase activity of point mutants in this residue: it was completely abolished, also in the case of the double mutant F389G/R387K, something that is specially noticeable since this enzyme also contains the R387K mutation that, alone, boosts terminal transferase activity by 100-fold (3).

In a second approach to study this ‘Loop1 network’, we mutated the conserved residues in a mini-loop (NSH motif) located in the thumb subdomain. In TdT, this mini-loop (DNH motif) is interacting with Loop1 through His^475^, conserved in Polμ (His^459^). This mini-loop is also present in the other members of the X family, but its function is different: residues from this loop directly interact with the template strand. In Polμ the role of this loop is dual: depending on the substrate used and the desired conformation of Loop1, this mini-loop may interact with the template strand (through Asn^457^) or with Loop1 (through His^459^). Accordingly, the asparagine is only needed during templated additions, and was dispensable for terminal transferase activity of Polμ, whilst the histidine mutation had the opposite effect. We propose a regulatory function for the NSH motif of the thumb mini-loop in Polμ: helping to accommodate either the template strand (as in Polß of Polλ) or Loop1 (as in TdT) as suits best for each individual situation.

Regarding the downstream template binding, Polμ has a positively charged helix that holds the phosphate backbone by means of four arginine residues. Of this four positive charges, only two are conserved in Polλ or Polß, which contribute to fidelity via their interaction with the template of the nascent base pair (23–26). In Polλ, these residues seem to be involved in controlling the motion of part of the thumb occurring during the transition from the binary (E:DNA) to the ternary (E:DNA:dNTP) complex (®-strand 8), which has the pursued effect of bringing the template strand closer to the thumb into its final catalytic position (22,30). A proposal that these residues play a similar role in Polμ was initially supported by molecular dynamics simulations (27). Our results further confirm this hypothesis, both using substrates with continuous template such as DNA gaps, which are correctly configured on their own, but specifically and more drastically during stabilization of two DNA ends during NHEJ. Our results indicate that these arginines do not contribute too much to general binding to the downstream end, given that the presence of the 5′-P group will provide the binding strength, but most likely these arginines are implicated in the bridging and positioning of the DNA substrates in a proper register to maximize efficiency and fidelity during NHEJ by Polμ. Based on our results with single-stranded substrates we are able to propose a second function for this arginine-containing helix: by establishing indirect interactions with the thumb mini-loop, it could be improving the stabilization of Loop1 in the case of maximal closure, i.e. during terminal transferase addition of nucleotides as well as during NHEJ reactions involving non-complementary ends. In this last scenario the involvement of the arginine helix could be dual, mediating protein–protein as well as protein–DNA interactions. This idea is further supported by new rounds of molecular dynamics in which these residues were individually mutated to alanine (27): these simulations, in the first place, displayed a conformation of the template strand similar to that observed for the binary complex in Polλ, demonstrating *in silico* the effect of these positive charges in pulling the downstream part of the substrate to its correct position; secondly, the final distance between Loop1 (modelled for the simulations) and the arginine helix is unusually large, and the former is in a conformation that may hamper its interactions with the DNA substrate.

Thus, having already discussed the roles of Loop1, the thumb mini-loop and the arginine helix, during untemplated additions and also during catalysis on discontinuous substrates, it seems now clear that these three portions of Polμ work coordinately, and together with another residue, His^329^, are involved in the terminal transferase and end-joining mechanisms (3,10). In Polμ, Loop1 is considered to be a highly flexible piece that could eventually adopt an indefinite number of conformations, but we argue that this is not so. At the time this manuscript was being finished for publication, a new set of crystal structures of human Polμ, including the apoenzyme, was obtained (29), This last one, although not showing the full conformation of Loop1 due to its flexibility, indicates that it would be anchored close to the active site, in a manner similar to that of TdT Loop1, where it would preclude binding of a template strand. The movements of His^329^ throughout the catalytic cycle can also be observed in these structures, and they are in agreement with our predictions (3), i.e. His^329^ points towards the active site only when primer and incoming nucleotide are present, whereas in the apoenzyme and pre- and post-catalytic binary complexes (with gaped and nicked DNA, respectively), this residue is oriented outwards and is not establishing any interactions via the side chain. The apoenzyme structure also fully supports our current findings since Phe^385^ and even His^381^ from Loop1 are found to be interacting with the thumb mini-loop. These interactions explain the dual phenotype observed with our NSH motif mutants: Asn^457^ is very important for NHEJ since it can interact with both His^381^ in Loop1 and Arg^445^ in the arginine helix, being the central connector of this network. His^459^, on the other hand, is mainly interacting with residues in Loop1 but not in the arginine helix, and thus its mutation would partially affect only one side of the network, primarily needed for untemplated additions.

**Figure 6.**
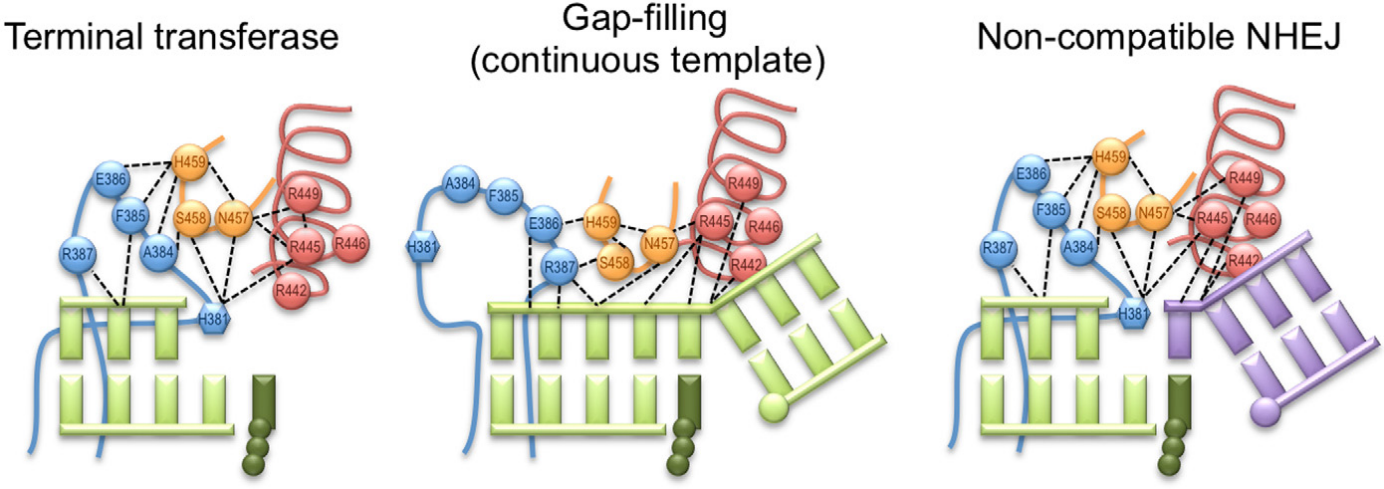
The ‘Loop1 network’: regulating the terminal transferase and NHEJ activities of Polμ through correct positioning of Loop1. Cartoons representing the network of human Polμ residues involved in positioning Loop1 during terminal transferase additions (left), gap filling (middle) and non-complementary NHEJ (right). Loop1 is shown in blue (His^381^ is shown as a hexagon), the thumb mini-loop is shown in orange, the arginine helix is shown in red, the incoming nucleotide is shown in dark green and the DNA substrates are shown in light green and mauve. Electrostatic interactions are shown as black dashed lines.

Based on all these data, we propose that Loop1 would maintain a fixed (TdT-like) initial position in a binary complex in which the DNA substrate (either ssDNA or a double-stranded DNA end containing long [+3 nt] 3′-protrusions and no 5′-phosphate group) is extended via untemplated insertions; in this case His^381^ could even act as a pseudo-template residue to help stabilize the primer terminus (Figure 6, left panel). In contrast, when Polμ binds a substrate containing a continuous template strand (i.e. a gap), Loop1 would become more disordered. This would probably be due to the new Loop1–NSH motif interaction (Glu^386^-His^459^) substituting that in the apoenzyme (Phe^385^-His^459^) and to the labile nature of other interactions keeping it in place (Figure 6, middle panel). A similar transition occurs even in the case of the smaller Loop1 present in Polλ: as dNTP binding induces the transition from binary to a ternary complex, β-strands β3 and β4 partially unravel to form Loop1, a nine-residue loop that repositions as the DNA template strand assumes its active conformation (30). In fact, one of the initial ß-strands is occupying the path that is filled by the template strand in the ternary complex. Finally, when Polμ binds two 3′-protruding NHEJ substrates, Loop1 needs to adopt a third conformation of inherent flexibility, to accommodate and stabilize the several possible locations and lengths of the gap formed in the template strand after synapsis. Direct protein–protein interactions with the thumb mini-loop, and indirect ones with the arginine helix, would assist Loop1 to be positioned in the correct orientation needed in each case (Supplementary Figure S3). In the specific case of non-complementary ends that form 1-nt gaps after synapsis, this network would be of special importance to keep the discontinuous template strand in frame, as shown in Figure 6 (right panel). Binding of the downstream end (through the 5′-P group) would cause the rearrangement of the arginines in helix N, and both Arg^449^ and Arg^445^ would be in a perfect position to contact the thumb mini-loop at Asn^457^. These three residues would be in charge of stabilizing the 1-nt gap formed in the template strand, whilst His^459^ and Ser^458^ would contact Loop1 to keep it in the right conformation.

Full understanding of the mechanisms of Loop1 conformational change and the alternative networks implicated in substrate recognition and end-bridging for decision-making during NHEJ will require new structural data of Polμ in complex with NHEJ substrates.

## SUPPLEMENTARY DATA

Supplementary Data are available at NAR Online.

SUPPLEMENTARY DATA
