# Supplementary material for: Decision-making during NHEJ: a network of interactions in human Polμ implicated in substrate recognition and end-bridging
Source: Nucleic Acids Res. 2014 May 31;42(12):7923–34. doi: 10.1093/nar/gku475 (PMC4081086; doi:10.1093/nar/gku475)
Supplement: SUPPLEMENTARY DATA [file supp_42_12_7923__index.html]

Decision-making during NHEJ: a network of interactions in human Polμ implicated in substrate recognition and end-bridging — Decision-making during NHEJ: a network of interactions in human Polμ implicated in substrate recognition and end-bridging — SUPPLEMENTARY DATA 

# Decision-making during NHEJ: a network of interactions in human Polμ implicated in substrate recognition and end-bridging

## SUPPLEMENTARY DATA

**Files in this Data Supplement:**

- SUPPLEMENTARY DATA
