# Supplementary material for: Decision-making during NHEJ: a network of interactions in human Polμ implicated in substrate recognition and end-bridging
Source: Nucleic Acids Res. 2014 May 31;42(12):7923–34. doi: 10.1093/nar/gku475 (PMC4081086; doi:10.1093/nar/gku475)
Supplement: SUPPLEMENTARY DATA [file supp_gku475_nar-00417-z-2014-File008.pdf]

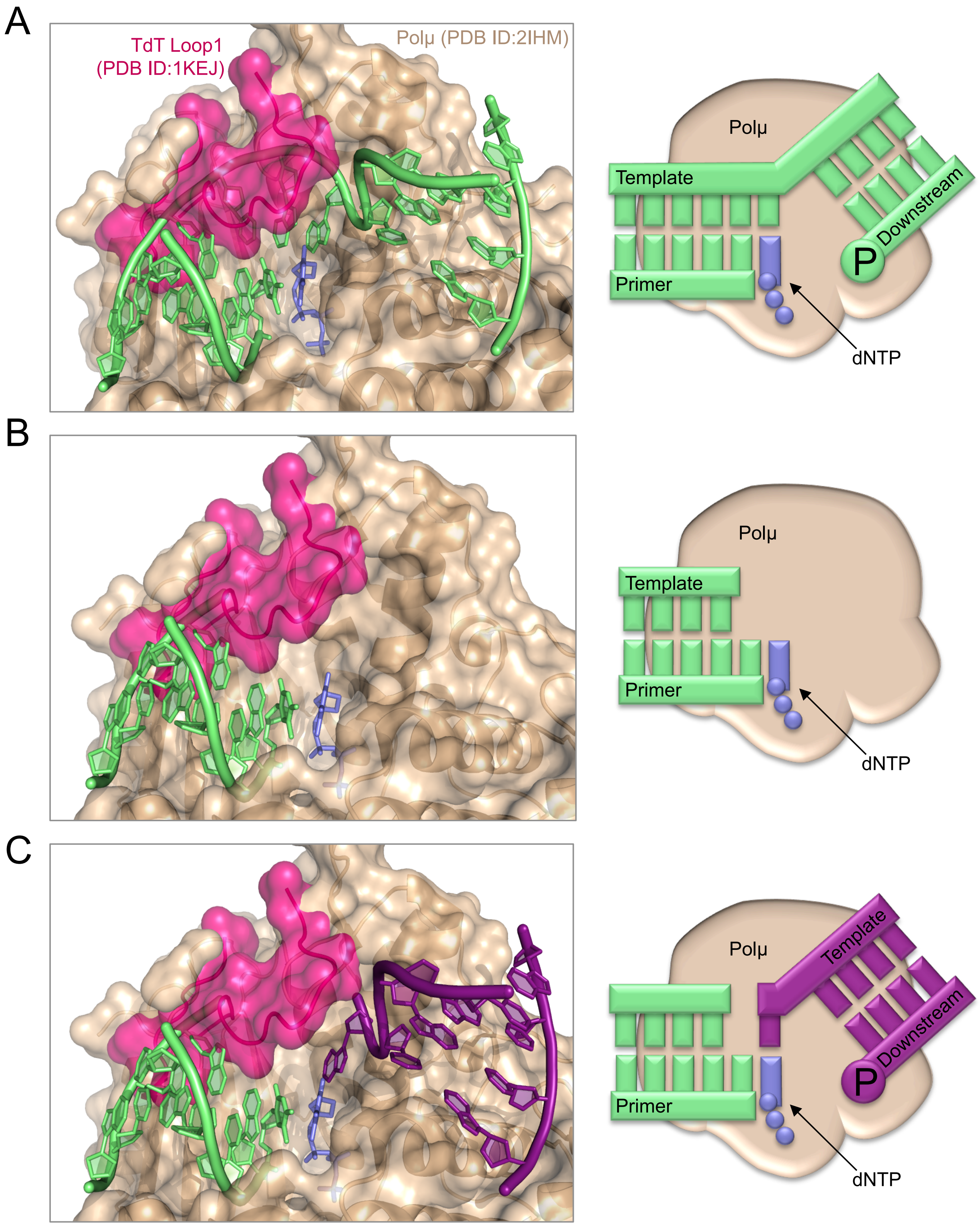
A
TdT Loop1
(PDB ID:1KEJ)
Polμ (PDB ID:2IHM)
Polμ
Template
Downstream
P
Primer
dNTP
B
Polμ
Template
Primer
dNTP
C
Polμ
Template
Downstream
P
Primer
dNTP

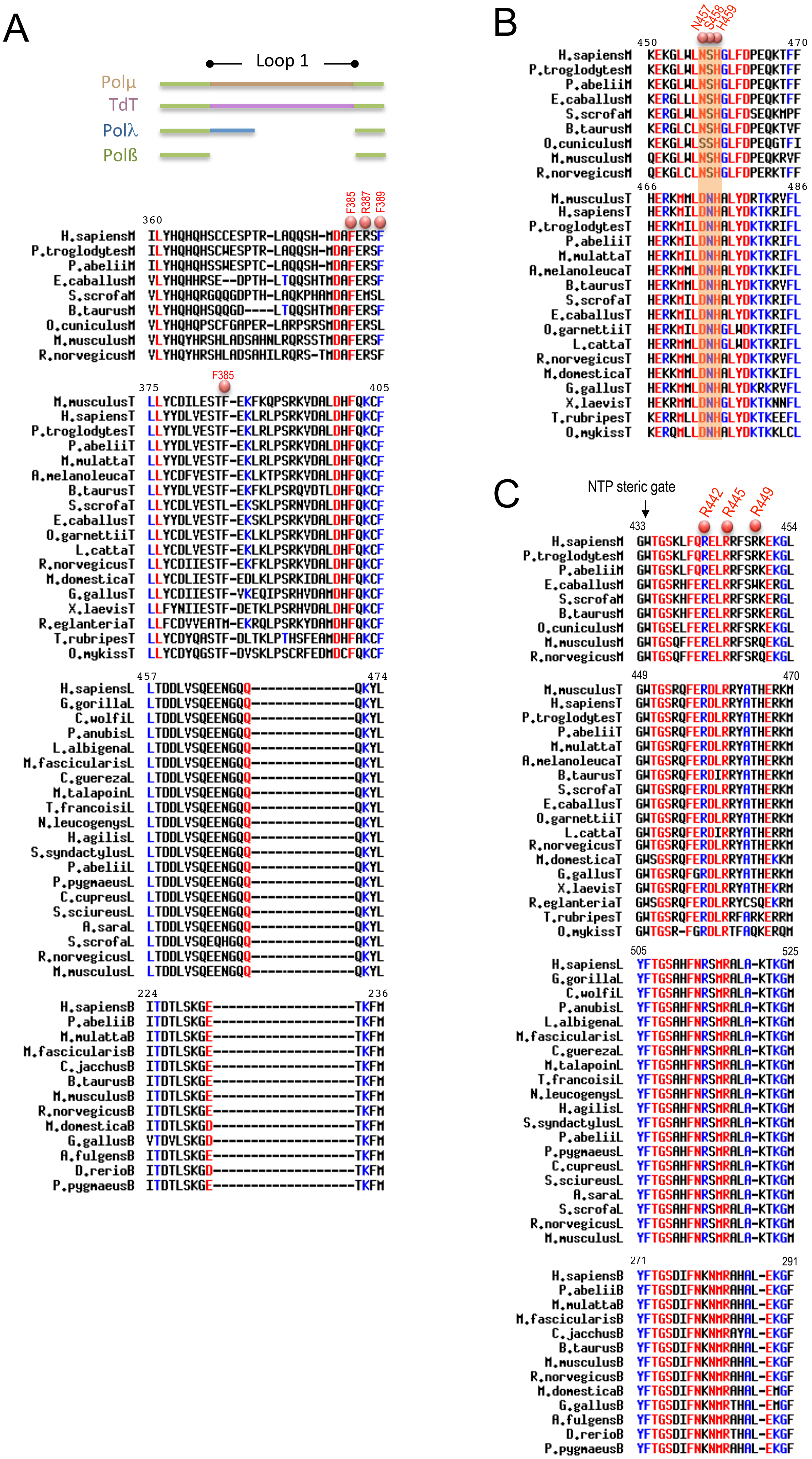
A
Loop 1
Polμ
TdT
Polλ
Polß
F385 R387 F389
360
H.sapiensM ILYHQHQHSCCESPTR-LAQQSH-MDAFERSF
P.troglodytesM ILYHQHQHSCWESPTR-LAQQSH-MDAFERSF
P.abeliiM ILYHQHQHSSWESPTC-LAQQSH-MDAFERSF
E.caballusM VLYHQHHRSE--DPTH-LTQQSHTMDAFERSF
S.scrofaM VLYHQHQRGQQGDPTH-LAQKPHAMDAFEMSL
B.taurusM VLYHQHQHSQQGD----LTQQSHTMDAFERSF
O.cuniculusM VLYHQHQPSCFGAPER-LARPSRSMDAFERSL
M.musculusM VLYHQYHRSHLADSAHNLRQRSSTMDAFERSF
R.norvegicusM VLYHQYHRSHLADSAHILRQRS-TMDAFERSF
F385
375 405
M.musculusT LLYCDILESTF-EKFKQPSRKYDALDHFQKCF
H.sapiensT LLYYDLVESTF-EKLRLPSRKVDALDHFQKCF
P.troglodytesT LLYYDLVESTF-EKLRLPSRKVDALDHFQKCF
P.abeliiT LLYYDLVESTF-EKLRLPSRKVDALDHFQKCF
M.mulattaT LLYYDLVESTF-EKLRLPSRKVDALDHFQKCF
A.melanoleucaT LLYCDFVESTF-EKLKTPSRKVDALDHFQKCF
B.taurusT LLYYDLVESTF-EKFKLPSRQVDTLDHFQKCF
S.scrofaT LLYCDLVESTL-EKSKLPSRNVDALDHFQKCF
E.caballusT LLYYDLVESTF-EKSKLPSRKVDALDHFQKCF
O.garnettiiT LLYCDLVESTF-EKLKLPSRKVDALDHFQKCF
L.cattaT LLYCDLVESTF-EKLKLPSRKVDALDHFQKCF
R.norvegicusT LLYCDIIESTF-EKFKLPSRKVDALDHFQKCF
M.domesticaT LLYCDLIESTF-EDLKLPSRKIDALDHFQKCF
G.gallusT LLYCDIIESTF-VKEQIPSRHVDAMDHFQKCF
X.laevisT LLFYNIIESTF-DETKLPSRHVDALDHFQKCF
R.eglanteriaT LLFCDVVEATM-EKRQLPSRKYDAMDHFQKCF
T.rubripesT LLYCDYQASTF-DLTKLPTHSFEAMDHFAKCF
O.mykissT LLYCDYQGSTF-DVSKLPSCRFEDMDCFQKCF
457 474
H.sapiensL LTDDLVSQEENGQQ--------------QKYL
G.gorillaL LTDDLVSQEENGQQ--------------QKYL
C.wolfiL LTDDLVSQEENGQQ--------------QKYL
P.anubisL LTDDLVSQEENGQQ--------------QKYL
L.albigenaL LTDDLVSQEENGQQ--------------QKYL
M.fascicularisL LTDDLVSQEENGQQ--------------QKYL
C.guerezaL LTDDLVSQEENGQQ--------------QKYL
M.talapoinL LTDDLVSQEENGQQ--------------QKYL
T.francoisiL LTDDLVSQEENGQQ--------------QKYL
N.leucogenysL LTDDLVSQEENGQQ--------------QKYL
H.agilisL LTDDLVSQEENGQQ--------------QKYL
S.syndactylusL LTDDLVSQEENGQQ--------------QKYL
P.abeliiL LTDDLVSQEENGQQ--------------QKYL
P.pygmaeusL LTDDLVSQEENGQQ--------------QKYL
C.cupreusL LTDDLVSQEENGQQ--------------QKYL
S.sciureusL LTDDLVSQEENGQQ--------------QKYL
A.saraL LTDDLVSQEENGQQ--------------QKYL
S.scrofaL LTDDLVSQEQHGQQ--------------QKYL
R.norvegicusL LTDDLVSQEENGQQ--------------QKYL
M.musculusL LTDDLVSQEENGQQ--------------QKYL
224 236
H.sapiensB ITDTLSKGE------------------TKFM
P.abeliiB ITDTLSKGE------------------TKFM
M.mulattaB ITDTLSKGE------------------TKFM
M.fascicularisB ITDTLSKGE------------------TKFM
C.jacchusB ITDTLSKGE------------------TKFM
B.taurusB ITDTLSKGE------------------TKFM
M.musculusB ITDTLSKGE------------------TKFM
R.norvegicusB ITDTLSKGE------------------TKFM
M.domesticaB ITDTLSKGD------------------TKFM
G.gallusB VTDVLSKGD------------------TKFM
A.fulgensB ITDTLSKGE------------------TKFM
D.rerioB ITDTLSKGD------------------TKFM
P.pygmaeusB ITDTLSKGE------------------TKFM
B
N457 S458 H459
450 470
H.sapiensM KEKGLWLNSHGLFDPEQKTFF
P.troglodytesM KEKGLWLNSHGLFDPEQKTFF
P.abeliiM KEKGLWLNSHGLFDPEQKTFF
E.caballusM KERGLLLNSHGLFDPEQKTFF
S.scrofaM KERGLWLNSHGLFDSEQKMPF
B.taurusM KERGLCLNSHGLFDPEQKTVF
O.cuniculusM KEKGLWLSSHGLFDPEQGTFI
M.musculusM QEKGLWLNSHGLFDPEQKRVF
R.norvegicusM QEKGLCLNSHGLFDPERKTFF
466 486
M.musculusT HERKMMLDNHALYDRTKRVFL
H.sapiensT HERKMILDNHALYDKTKRIFL
P.troglodytesT HERKMILDNHALYDKTKRIFL
P.abeliiT HERKMILDNHALYDKTKRIFL
M.mulattaT HERKMILDNHALYDKTKRIFL
A.melanoleucaT HERKMMLDNHALYDKTKKIFL
B.taurusT HERKMMLDNHALYDKTKRVFL
S.scrofaT HERKMILDNHALYDKTKRIFL
E.caballusT HERKMILDNHALYDKTKRIFL
O.garnettiiT HERKMILDNHGLWDKTKRIFL
L.cattaT HERRMMLDNHGLWDKTKRIFL
R.norvegicusT HERKMMLDNHALYDKTKRVFL
M.domesticaT HEKKMMLDNHALYDKTKKIFL
G.gallusT HERKMMLDNHALYDKRKRVFL
X.laevisT HEKRMMLDNHGLYDKTKNNFL
T.rubripesT KERRMLLDNHGLYDKTKEEFL
O.mykissT KERQMLLDNHALYDKTKKLCL
C
NTP steric gate
R442 R445 R449
433 454
H.sapiensM GWTGSKLFQRELRRFSRKEKGL
P.troglodytesM GWTGSKLFQRELRRFSRKEKGL
P.abeliiM GWTGSKLFQRELRRFSRKEKGL
E.caballusM GWTGSRHFERELRRFSWKERGL
S.scrofaM GWTGSKHFERELRRFSRKERGL
B.taurusM GWTGSKHFERELRRFSRKERGL
O.cuniculusM GWTGSELFERELRRFSRKEKGL
M.musculusM GWTGSQFFERELRRFSRQEKGL
R.norvegicusM GWTGSQFFERELRRFSRQEKGL
449 470
M.musculusT GWTGSRQFERDLRRYATHERKM
H.sapiensT GWTGSRQFERDLRRYATHERKM
P.troglodytesT GWTGSRQFERDLRRYATHERKM
P.abeliiT GWTGSRQFERDLRRYATHERKM
M.mulattaT GWTGSRQFERDLRRYATHERKM
A.melanoleucaT GWTGSRQFERDLRRYATHERKM
B.taurusT GWTGSRQFERDIRRYATHERKM
S.scrofaT GWTGSRQFERDLRRYATHERKM
E.caballusT GWTGSRQFERDLRRYATHERKM
O.garnettiiT GWTGSRQFERDLRRYATHERKM
L.cattaT GWTGSRQFERDIRRYATHERRM
R.norvegicusT GWTGSRQFERDLRRYATHERKM
M.domesticaT GWSGSRQFERDLRRYATHEKKM
G.gallusT GWTGSRQFGRDLRRYATHERKM
X.laevisT GWTGSRQFERDLRRYATHEKRM
R.eglanteriaT GWSGSRQFERDLRRYCSQEKRM
T.rubripesT GWTGSRQFERDLRRFARKERRM
O.mykissT GWTGSR-FGRDLRTFAQKERQM
505 525
H.sapiensL YFTGSAHFNRSMRALA-KTKGM
G.gorillaL YFTGSAHFNRSMRALA-KTKGM
C.wolfiL YFTGSAHFNRSMRALA-KTKGM
P.anubisL YFTGSAHFNRSMRALA-KTKGM
L.albigenaL YFTGSAHFNRSMRALA-KTKGM
M.fascicularisL YFTGSAHFNRSMRALA-KTKGM
C.guerezaL YFTGSAHFNRSMRALA-KTKGM
M.talapoinL YFTGSAHFNRSMRALA-KTKGM
T.francoisiL YFTGSAHFNRSMRALA-KTKGM
N.leucogenysL YFTGSAHFNRSMRALA-KTKGM
H.agilisL YFTGSAHFNRSMRALA-KTKGM
S.syndactylusL YFTGSAHFNRSMRALA-KTKGM
P.abeliiL YFTGSAHFNRSMRALA-KTKGM
P.pygmaeusL YFTGSAHFNRSMRALA-KTKGM
C.cupreusL YFTGSAHFNRSMRALA-KTKGM
S.sciureusL YFTGSAHFNRSMRALA-KTKGM
A.saraL YFTGSAHFNRSMRALA-KTKGM
S.scrofaL YFTGSAHFNRSMRALA-KTKGM
R.norvegicusL YFTGSAHFNRSMRALA-KTKGM
M.musculusL YFTGSAHFNRSMRALA-KTKGM
271 291
H.sapiensB YFTGSDIFNKNMRAHAL-EKGF
P.abeliiB YFTGSDIFNKNMRAHAL-EKGF
M.mulattaB YFTGSDIFNKNMRAHAL-EKGF
M.fascicularisB YFTGSDIFNKNMRAHAL-EKGF
C.jacchusB YFTGSDIFNKNMRAYAL-EKGF
B.taurusB YFTGSDIFNKNMRAHAL-EKGF
M.musculusB YFTGSDIFNKNMRAHAL-EKGF
R.norvegicusB YFTGSDIFNKNMRAHAL-EKGF
M.domesticaB YFTGSDIFNKNMRAHAL-EMGF
G.gallusB YFTGSDIFNKNMRTHAL-EMGF
A.fulgensB YFTGSDIFNKNMRAHAL-EKGF
D.rerioB YFTGSDIFNKNMRTHAL-EKGF
P.pygmaeusB YFTGSDIFNKNMRAHAL-EKGF

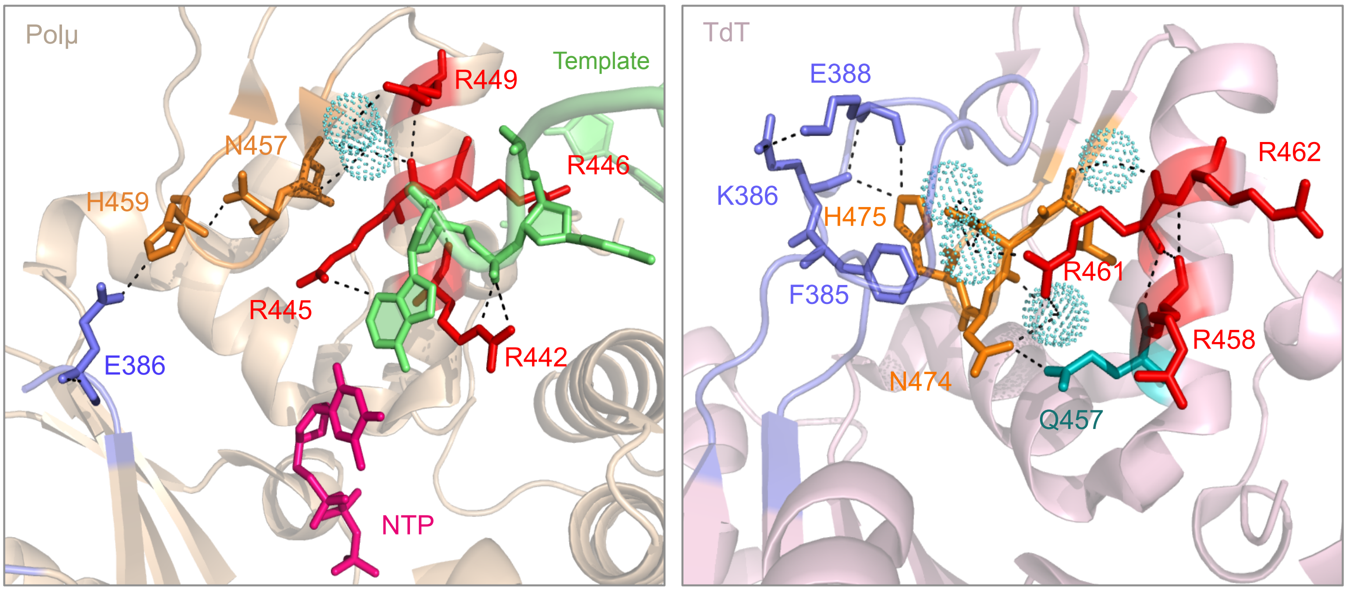
Polμ
Template
R449
N457
R446
H459
R445
R442
E386
NTP
TdT
E388
R462
K386
H475
R461
F385
R458
N474
Q457

Supplementary figure 1. A to C) Superimposition of Loop1 from the crystal of the TdT apoenzyme (PDB ID: 1JMS), shown in dark pink cartoon and semi-transparent surface, on the murine Polμ ternary complex structure (PDB ID: 2IHM), shown as a wheat-coloured surface. A) The DNA substrate (1 nt gap) is shown in green cartoon, and the incoming dNTP in blue sticks. B) The original DNA substrate has been trimmed to show a 1 nt 3'-protruding template/primer structure (green cartoon). C) The original DNA substrate has been trimmed to show two 1 nt 3'-protruding structures (template/primer: green cartoon; template/downstream: purple cartoon). In each of the three panels (A-C), a cartoon on the right hand side depicts a schematic of the complex shown in the structure to the left.

Supplementary figure 2. Multiple amino acid sequence alignment showing the regions corresponding to: A) Loop1; B) the NSH motif (thumb mini-loop); C) the arginine helix, with a different degree of conservation among Polμ (M), TdT (T), Polλ (L) and Polß (B) from different species. Spheres indicate Polμ selected residues that have been mutated in this study (red), or their murine TdT counterparts. Invariant residues are shown in red, highly conserved residues in blue.

Supplementary figure 3. The "Loop1 network": regulating the terminal transferase and NHEJ activities of Polμ through correct positioning of Loop1. Cartoon representations of the ternary complex of murine Polμ (2IHM, left panel) and the murineTdT apoenzyme (1JMS, right panel). Loop 1 is shown in blue cartoon with selected residues involved in interactions shown in sticks; the thumb mini-loop is shown in orange with selected residues shown in sticks; arginines from the helix N are shown in red sticks; water molecules and other residues involved in the network of interactions are shown in light teal. In the case of Polμ, the incoming nucleotide is shown in dark pink and the template strand is shown in green. Numbering of Polμ residues corresponds to the human enzyme.
